# Prenatal alcohol exposure increases the susceptibility to develop aggressive prolactinomas in the pituitary gland

**DOI:** 10.1038/s41598-018-25785-y

**Published:** 2018-05-16

**Authors:** Shaima Jabbar, Kenneth Reuhl, Dipak K. Sarkar

**Affiliations:** 1 The Endocrine Program, Department of Animal Sciences, Rutgers, The State University of New Jersey, 67 Poultry Lane, New Brunswick, NJ 08901 USA; 2 Endocrinology and Animal Biosciences Graduate Program, Rutgers, The State University of New Jersey, 84 Lipman Drive, New Brunswick, NJ 08901 USA; 3 The Environmental and Occupational Health Sciences Institute, Department of Pharmacology and Toxicology, Ernest Mario School of Pharmacy, Rutgers, The State University of New Jersey, 140, Frelinghuysen Road, Piscataway, NJ 08854 USA

## Abstract

Excess alcohol use is known to promote development of aggressive tumors in various tissues in human patients, but the cause of alcohol promotion of tumor aggressiveness is not clearly understood. We used an animals model of fetal alcohol exposure that is known to promote tumor development and determined if alcohol programs the pituitary to acquire aggressive prolactin-secreting tumors. Our results show that pituitaries of fetal alcohol-exposed rats produced increased levels of intra-pituitary aromatase protein and plasma estrogen, enhanced pituitary tissue growth, and upon estrogen challenge developed prolactin-secreting tumors (prolactinomas) that were hemorrhagic and often penetrated into the surrounding tissue. Pituitary tumors of fetal alcohol-exposed rats produced higher levels of hemorrhage-associated genes and proteins and multipotency genes and proteins. Cells of pituitary tumor of fetal alcohol exposed rat grew into tumor spheres in ultra-low attachment plate, expressed multipotency genes, formed an increased number of colonies, showed enhanced cell migration, and induced solid tumors following inoculation in immunodeficient mice. These data suggest that fetal alcohol exposure programs the pituitary to develop aggressive prolactinoma after estrogen treatment possibly due to increase in stem cell niche within the tumor microenvironment.

## Introduction

The National Cancer Institute has established that alcoholic beverage is a human teratogen and human carcinogen^1^. Moreover, International Association of Research on Cancer’s 2009 survey identify that around 3.5% of cancer deaths in USA are caused by alcohol consumption^2,3^. A number of reports have now demonstrated that alcohol abuse promotes development of aggressive tumors of breast, prostate, pancreas, and colon tissues in human patients^4–6^. The cause of alcohol promotion of tumor aggressiveness is not clearly understood, but recent evidence suggests that enhanced estrogen signaling might be involved in alcohol promotion of tumor aggressiveness. Using an animal model of fetal alcohol exposure, Hilakivi-Clarke and colleagues have shown elevated plasma estrogen levels may increase the risk for mammary tumor development in fetal alcohol exposed offspring^7^. Polanco and colleagues have also connected increased plasma estrogen levels with the tumor promotion in fetal alcohol exposed rats^8,9^. The ovarian steroid estradiol is also known to increase pituitary prolactin (PRL)-producing lactotropic cell proliferation and to induce prolactinoma development in humans as well as in laboratory animals^10–14^. Prolactinomas account for approximately 40% of all pituitary tumors and are an important cause of hypogonadism and infertility in human^15,16^. While most are benign and can be controlled by current therapies, some show a lack of sensitivity to a combination therapies or recur during follow-up and are considered as aggressive with unclear epidemiology^17^. Whether environmental imprinting may influence the development of aggressive prolactinomas has not been studied. We therefore determined if fetal alcohol exposure, which causes stable alcohol epigenetic marks on the genome^18–20^, promotes development of aggressive prolactinomas using a Fischer-344 (F344) rat animal model.

## Results

### Fetal alcohol exposure increases pituitary weights and levels of pituitary prolactin (PRL), plasma PRL, pituitary aromatase, pituitary ESR1 and plasma estrogen in female offspring

**Figure 1:**
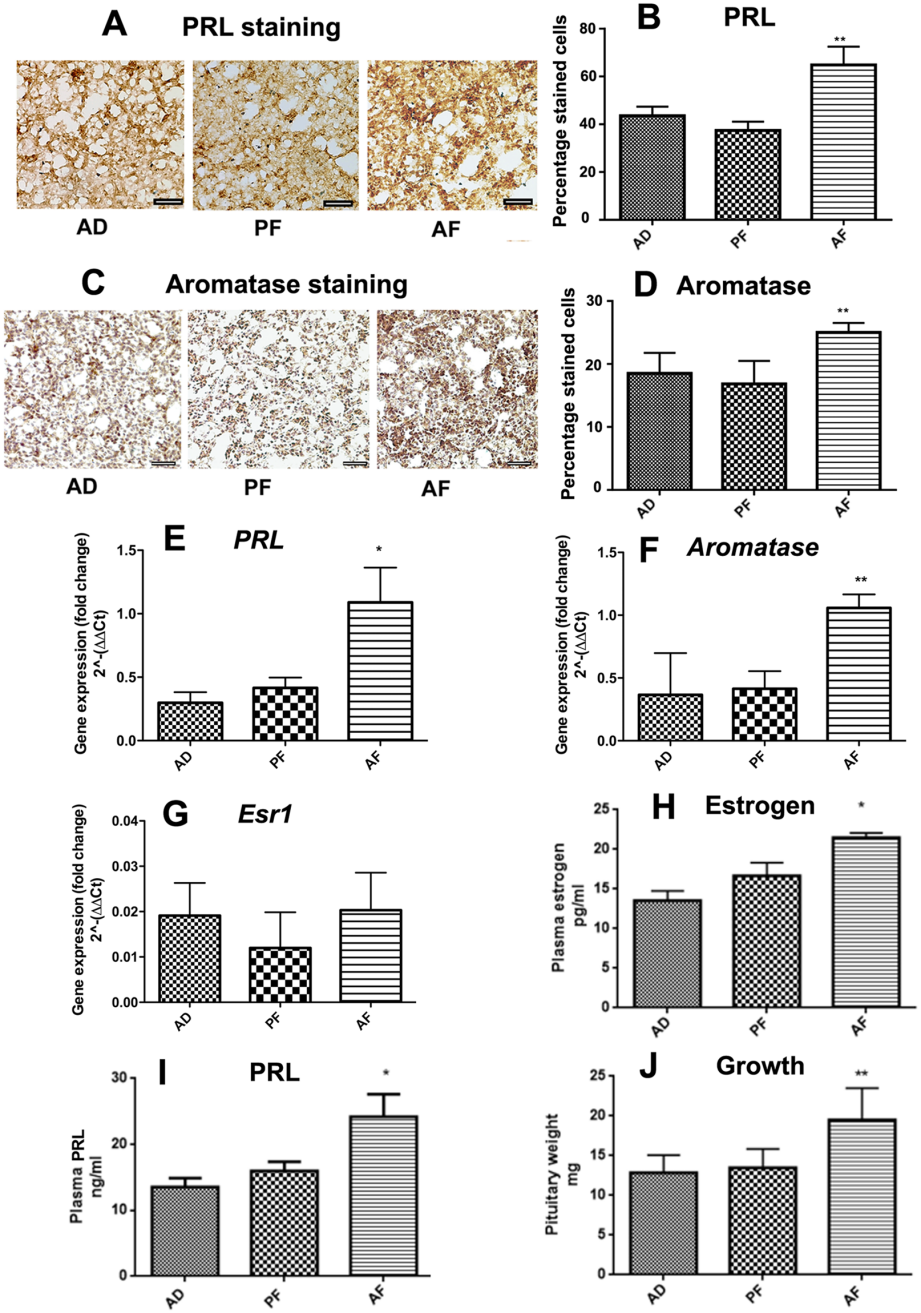
Effects of fetal alcohol exposure on pituitary prolactin (PRL) content (**A**,**B**) and aromatase content (**C**,**D**) as determined by immunocytochemical detection, pituitary *Prl* mRNA level (**E**), aromatase mRNA level (**F**), plasma estrogen level (**G**), pituitary estrogen receptor 1 (*Esr1*) mRNA level (**H**) plasma PRL (**I**) and pituitary weight (**J**) in female rats. Alcohol-fed (AF), pair-fed (PF) and ad libitum-fed (AD) rat offspring were used during the adult period (90 days) on the day of estrus. Data are mean ± SEM (n = 6–8) and were analyzed using one-way analysis of variance (ANOVA) with the Newman-Keul posthoc test. *p < 0.05, **p < 0.01 and ***p < 0.001 between AF and controls (AD, PF). Scale bar represents 100 µM.

In the first study, we evaluated if fetal alcohol exposure increased pituitary and systemic levels of estrogen and alters lactotrope functions by measuring the pituitary content of PRL and aromatase protein levels using immunohistochemical procedures. We also measured aromatase and alpha estrogen receptor mRNA levels in these tissues using quantitative RT-PCR methods. Additionally, we measured plasma levels of PRL and estrogen by ELISA assays and also determined pituitary weight differences between fetal alcohol-fed and control-fed rats. As expected from our previous work^21^, pituitary PRL immunoreactivity was increased in fetal alcohol-fed (AF) animals (Fig. 1A,B). Immunohistochemical staining data also showed increased numbers of aromatase-stained cells in AF animals’ pituitary tissues (Fig. 1C,D). We found that *Prl* mRNA and aromatase mRNA levels, but not estrogen receptor 1 (*Esr1*) mRNA levels, were elevated in AF offspring as compared to control-fed (AD and PF) offspring (Fig. 1E–G). Additionally, we found that plasma estrogen levels (Fig. 1H), plasma PRL levels (Fig. 1I) and pituitary weights (Fig. 1J) were higher in AF animals as compared to controls. These data suggest that in utero alcohol exposure enhances the production of plasma and pituitary estrogen and PRL levels and the pituitary weight during the adult period in the offspring.

### Fetal alcohol exposure increases the development of prolactinomas with aggressive behavioral phenotypes in the pituitary after estrogen treatment in adult female rats

**Figure 2:**
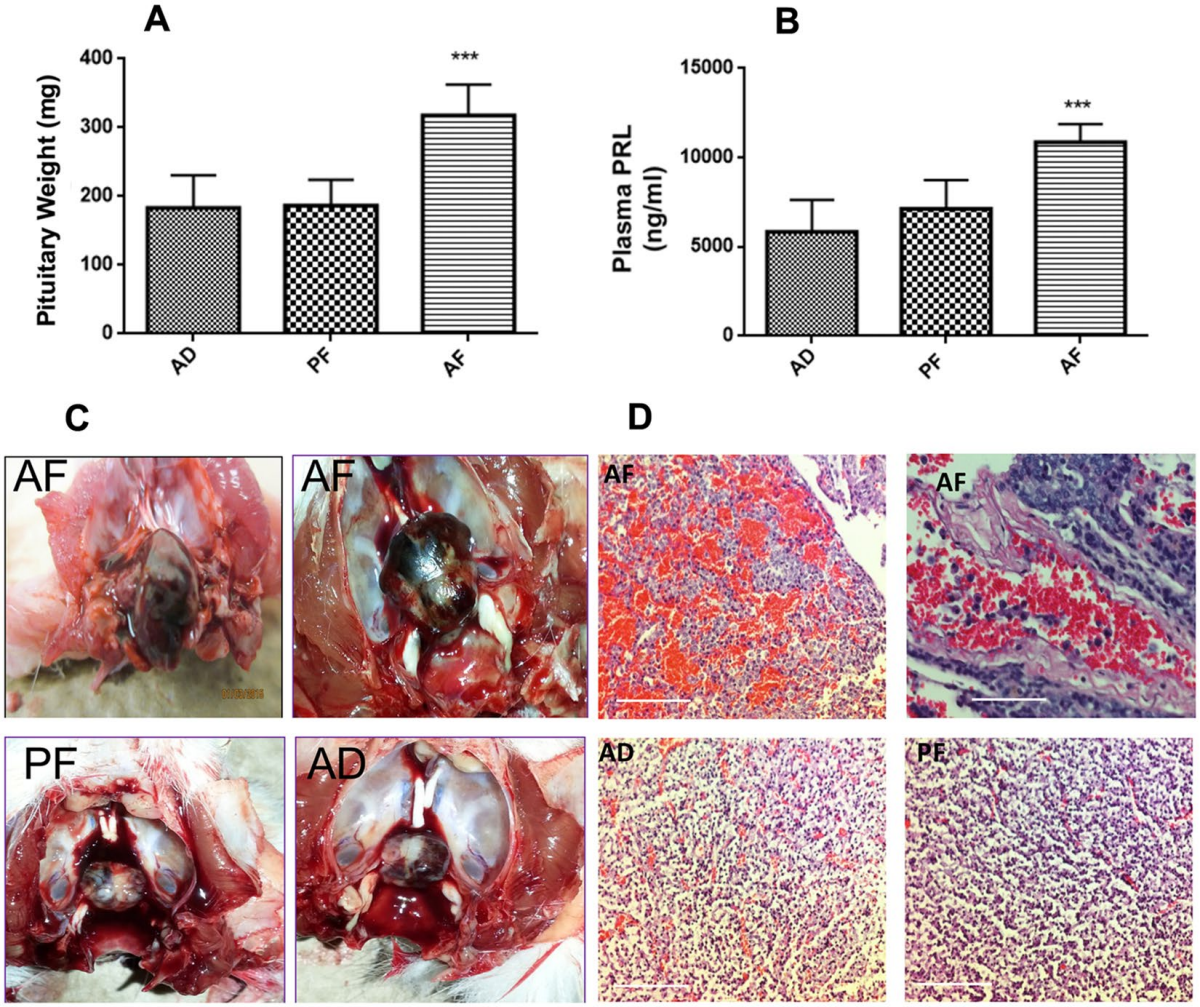
Effects of fetal alcohol exposure on estrogen-induced changes in pituitary weight (**A**), plasma PRL level (**B**) and pituitary histopathology (**C**). Alcohol-fed (AF) or control-fed (PF, AD) rats offspring were ovariectomized and implanted with a β-estradiol implants at 60 days of age and after 60 (E2-60 d), 90 days (E2-90 d) or 120 days (E2-120 d) were used for measuring pituitary weight and plasma PRL. Data are mean ± SEM (n = 6–8) and were analyzed using one-way analysis of variance (ANOVA) with the Newman-Keul posthoc test. *p < 0.05, **p < 0.01 and ***p < 0.001 between AF and controls (AD, PF). Histopathology of AF, PF or AD offspring pituitary is evaluated following histological staining using Hematoxylin and Eosin and microscopic evaluation by a pathologist Dr. Kenneth Reuhl. Representative photomicrographs of the pituitaries are shown. Scale bar represents 100 µM.

Because estrogen is mitogenic to lactotropes^12–14^, we hypothesized that prolonged elevation of estrogen production, particularly in the pituitary, in fetal alcohol exposed offspring may lead to the development of pituitary tumors. To address this, we first compared the effects of estrogen on pituitary weight and plasma PRL in fetal alcohol exposed and control diet-exposed offspring. These data are shown in Fig. 2. As demonstrated in this figure, estrogen-treatment via a silastic capsule known to maintain plasma estrogen levels about 150 pg/ml for a period of three months^22^, increased both pituitary weights (Fig. 2A) and plasma levels of PRL (Fig. 2B). Inspection of the pituitary gland 120 days after estrogen treatment revealed that some of the tumors in pituitaries of AF animals were highly vascularized and penetrated to the sphenoid bone (Fig. 2C; top two panels). Pituitaries of estrogen-treated AD and PF rats were smaller, less vascularized and were situated within the sphenoid bone (Fig. 2C; bottom two panels). Histopathological inspections showed that pituitaries of AF animals were composed of small round tumor cells in a solid pattern, densely granulated and strongly acidophilic (indicator of rare prolactinomas), with necrotic regions, the epithelial cells colonized in nested shape surrounded with blood vessels (indicators of angiogenesis signaling) (Fig. 2D; top two panels. The pituitary tumor section from AD and PF animals, in contrast, showed uniform, weakly acidophilic epithelial cells with infiltration of eosinophilic cells (Fig. 2D; bottom two panels). These data provide both anatomical and histopathological evidence for the development of aggressive type pituitary prolactin secreting tumors in fetal alcohol-exposed female rats following estrogen treatment.

### Fetal alcohol exposure increases expression of biomarkers for pituitary tumor aggressiveness

**Figure 3:**
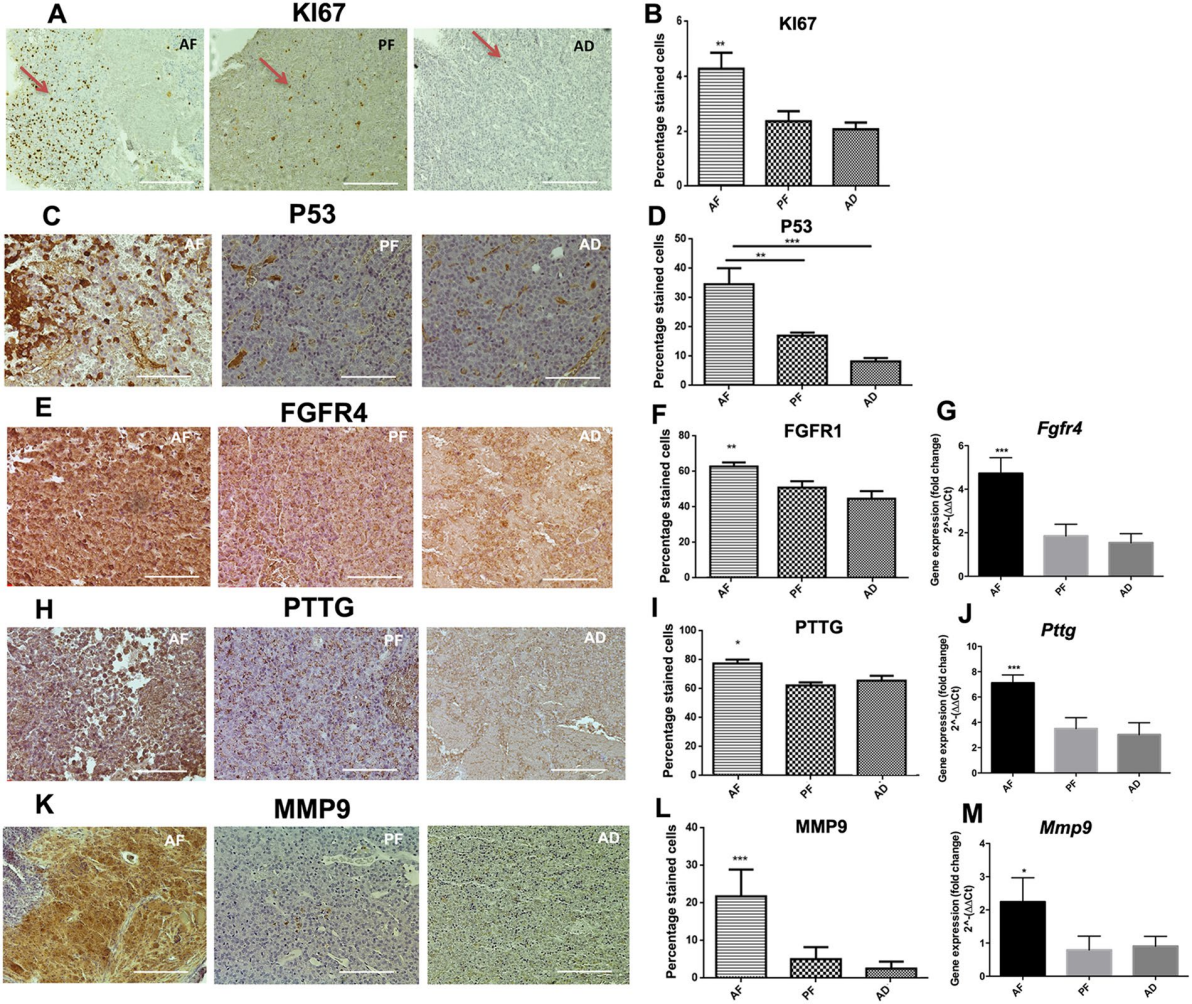
Characterization of the expression of tumor aggressiveness markers in the pituitary of fetal alcohol exposed offspring. Pituitary tumor tissues of AF, PF and AD rats were stained for Ki67, P53, FGF4, PTTG or MMP9 using histochemical techniques. Photomicrographs of these tumor aggressiveness markers are shown on the left panels (**A**,**C**,**E**,**H**,**K**) and the percentage of the positive staining represented on the right panels (**B**,**D**,**F**,**I**,**L**). Only the dark brown staining is used for counting the positive staining as described in the methods section. The magnification 10× for Ki67 (scale bar represents 100 µM) and 20× (scale bar represents 50 µM) for the rest (P53, FGF4, PTTG or MMP9). The expression levels of *Fgf4, Pttg* or *Mmp9* mRNAs are shown in panels G, J and M, respectively. Data are expressed as mean ± SEM (n = 6–8) and were analyzed using one-way analysis of variance (ANOVA) with the Tukey’s multiple comparisons posttest. *p < 0.05, **p < 0.01 and ***p < 0.001 between AF and controls (AD, PF) or indicated by a bar on the top of the graphs.

As noted earlier, pituitary tumors are generally adenomas but some may show aggressive and/or malignant behaviors^23^. Several biochemical markers (Ki67, P53, FGFR4, PTTG, MMP9) are used to identify the tumor aggressiveness in clinics^24^. By employing immunohistochemical methods we determined the changes in these biochemical marker proteins in pituitaries of fetal alcohol-fed (AF) and control-fed (AD and PF) rats. As shown in Fig. 3A,B, KI67 immuno-labelled cell number was higher in the pituitary of AF rat than those in the pituitary of AD and PF controls. Similarly, the number of P53 immuno-labelled cells was elevated in the pituitary of AF rats (Fig. 3C,D). The protein and mRNA levels of oncogenes (FGFR4, PTTG and MMP9) in the pituitaries were also determined. Fetal alcohol exposed pituitaries showed increased protein (Fig. 3E,F) and gene expression levels of FGFR4 (Fig. 3G) as compared to control rat pituitaries. Fetal alcohol-exposed pituitaries also showed increased protein and mRNA levels of PTTG (Fig. 3H–J) and MMP9 (Fig. 3K–M). These biochemical data are in agreement with those histopathological data shown in Fig. 2 and support the view that pituitaries of fetal alcohol exposed rats develop aggressive tumors following estrogen treatment.

### Fetal alcohol exposure increases expression of stem-like cells in the pituitary tumors

**Figure 4:**
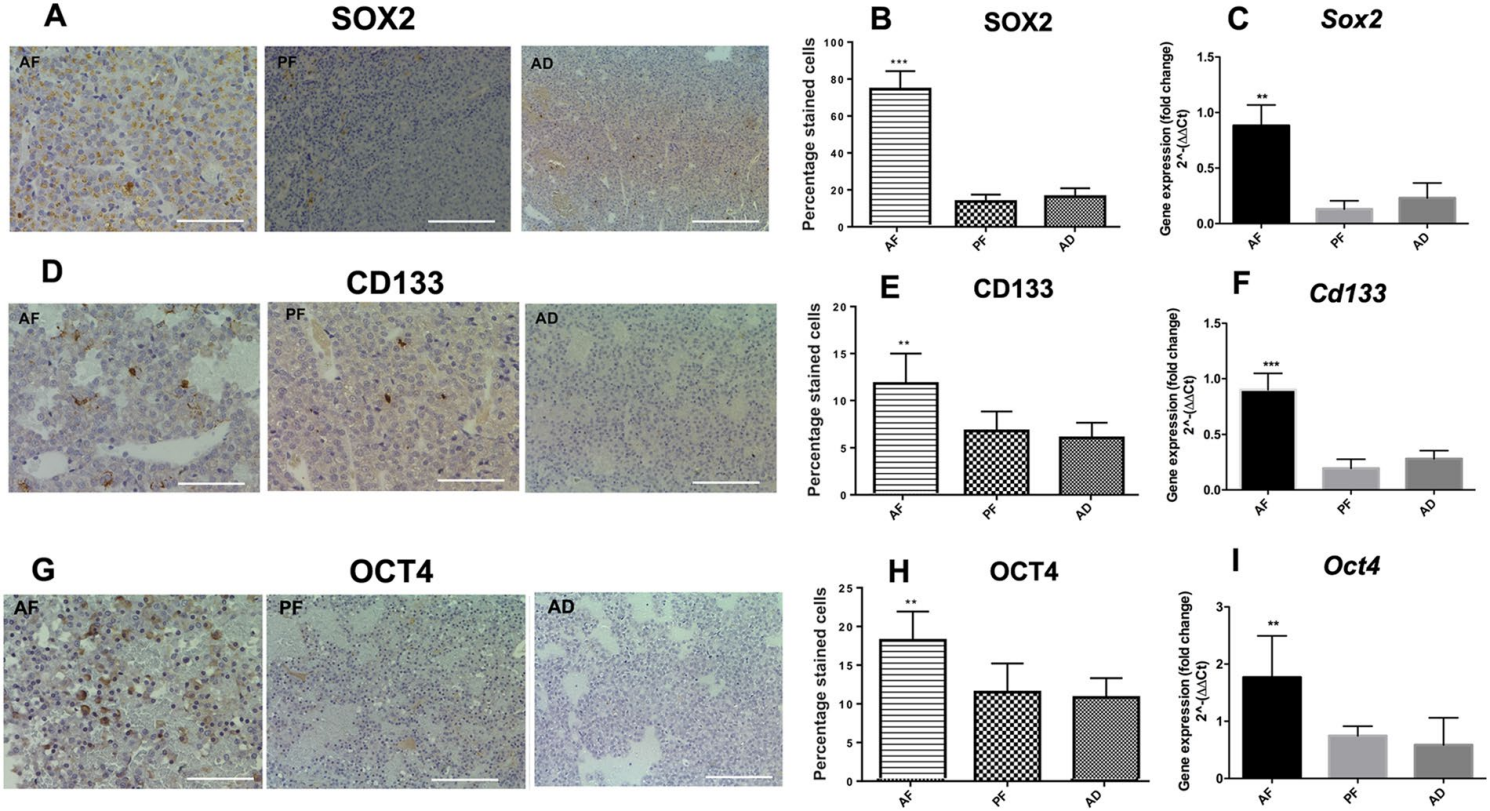
Expression of cancer stem cell (CSC) markers in the pituitary of fetal alcohol exposed offspring. Photomicrographs of these CSC markers are shown on the left panels (**A**,**D**,**G**) and the percentage of the positive staining represents on the right panels (**B**,**E**,**H**). Only the dark brown staining is used for counting the positive staining as described in the methods section. Scale bar represents 50 µM. The expression level of *Sox2, Oct4* or *Cd133* mRNAs are shown in panels C, F and I, respectively. Data are expressed as mean ± SEM (n = 6–8) and were analyzed using one-way analysis of variance (ANOVA) with the Tukey’s multiple comparisons posttest. *p < 0.05, **p < 0.01 and ***p < 0.001.

Recently, pituitary stem cells (PSCs) have been proposed to play a role in pituitary tumorigenesis, particularly in the formation of aggressive pituitary tumors^25,26^. To determine if pituitary tumors of fetal alcohol-exposed rats contains PSCs, we measured stem cell marker proteins SOX2, CD133 and OCT4 by immunocytochemistry and their precursor genes by q-PCR in the tumor tissues. As shown in Fig. 4, SOX2, CD133 and OCT4 immuno-labelled cell numbers were higher in pituitaries of fetal alcohol-exposed rats than those in pituitaries of control rats (Fig. 4A,B,D,E,G,H). Fetal alcohol-exposed pituitaries also showed increased mRNA levels of *Sox2* (Fig. 4C), *Cd133* (Fig. 4E) and *Oct4* (Fig. 4H) compared to control rat pituitaries. These data suggest that the tumors in the pituitary of fetal alcohol-exposed rats contain more stem-like cells than those in pituitaries of control rats.

**Figure 5:**
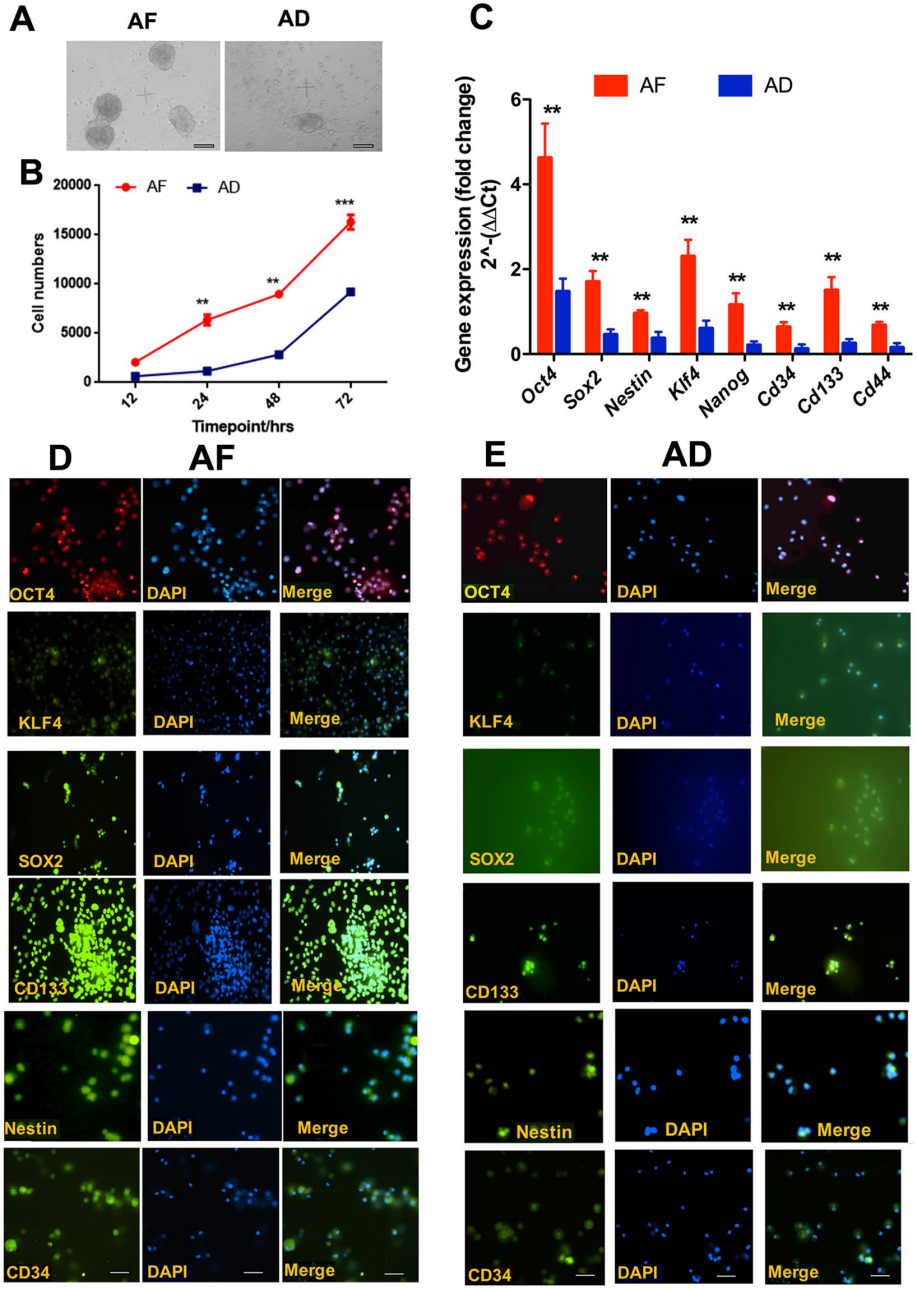
Characterization of pituitary tumor cell spheres (tumorispheres) isolated from the anterior part of pituitary tumor of fetal alcohol exposed (AF) or control (AD) rats. (**A**) Images of tumorispheres from AF and AD rats that were formed after 7 days in culture in ultralow attachment plate in serum free growth medium. (**B**) Showing the cell proliferation rate of tumorispheres from AF and AD rats in cultures. Data are expressed as mean ± SEM (n = 5–6) and were analyzed using one-way analysis of variance (ANOVA) with the Tukey’s multiple comparisons posttest. **p < 0.01 and ***p < 0.001 between AF and AD. (**C**) Expression levels of various stem cell marker genes in tumorispheres of AF and AD rats. (**D**) and (**E**) Representative photographs of immunofluorescent detection of stem cell-associated transcription factor proteins in AF rat (D) and AD rat’s tumorispheres. Scale bar represents 50 µM.

When pituitary tumor cells of AF-treated and control (AD)-treated rats were grown in ultra-low attachment plates, a large number of spheres developed from AF pituitary tumor cells while a small number of spheres developed from AD pituitary tumor cells (Fig. 5A). AF pituitary tumor cell spheres (tumorispheres) grew rapidly and at a six-fold higher rate than that in AD pituitary tumorispheres (Fig. 5B). AF tumorispheres expressed higher levels of stem cell marker (*Oct4, Nanog, Klf4, Sox2, Cd133, Cd44, Nestin, and Cd34*) mRNAs (Fig. 5C) and most of their protein counterparts (Fig. 5D). These results indicate that pituitary tumors of AF rats have expanded stem cell niche.

### Pituitary tumorisphere cells of fetal alcohol exposed rat show increased tumorigenicity

**Figure 6:**
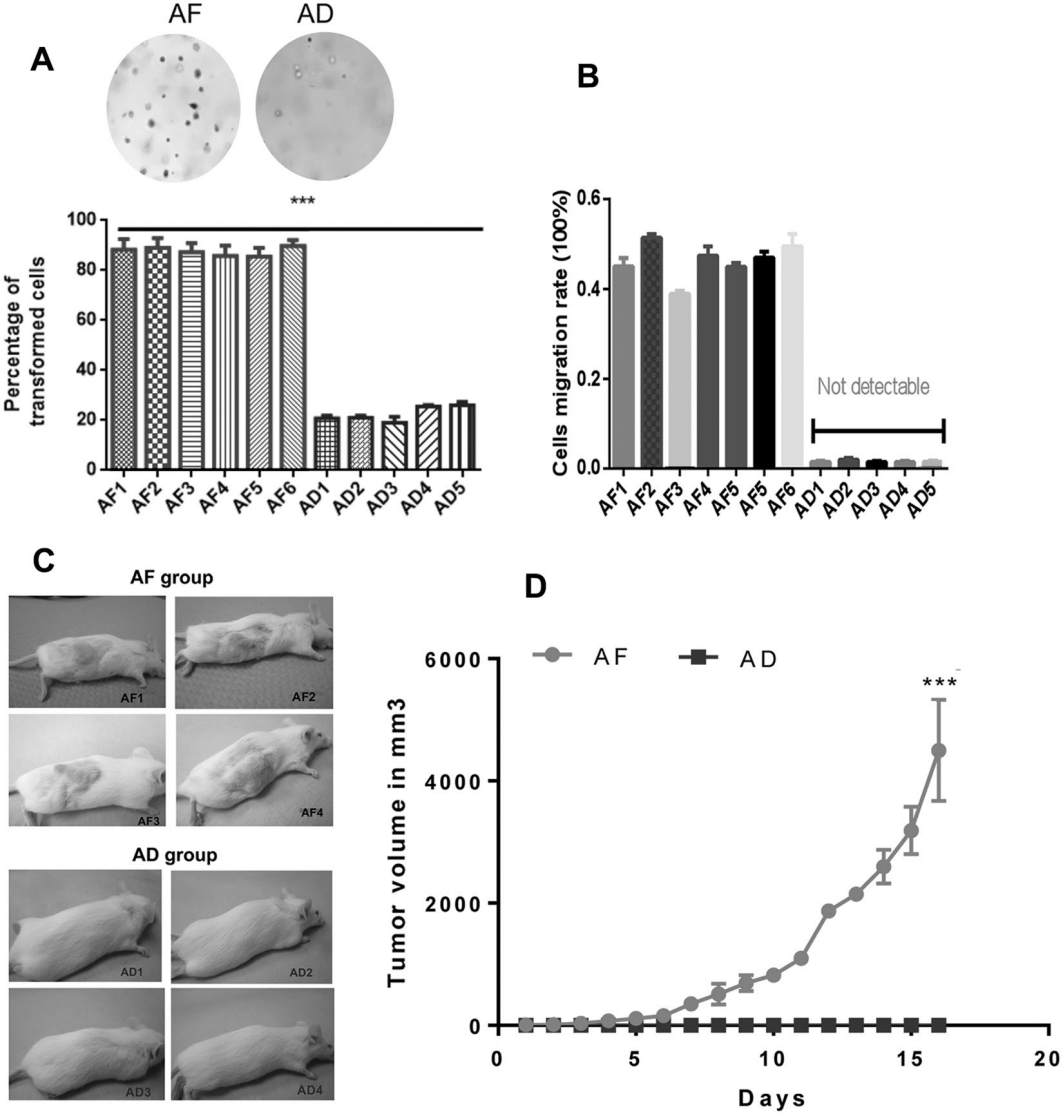
Determination of cell colony formation, migration and tumorigenic ability of tumorispheres of fetal alcohol exposed rats (AF) and control rats (AD). Tumorispheres were obtained from pituitary tumors of AF and AD treated rats and maintained in ultralow attachment plate with serum-free growth medium for several generations. (**A**) Showing the colony formation in soft agar plate of AF and AD tumorispheres (top) and the number of transformed cells counts (bottom). Data are mean ± SEM (n = 5) and analyzed using ANOVA with the with the Tukey’s multiple comparisons posttest. ***p < 0.001 between AF and AD. (**B**) Cell migration rate of AF and AD tumorispheres. Data are mean ± SEM (n = 3). (**C**) Representative photos of immunodeficient Nude/SCID mice injected with AF or AD tumorispheres (5 × 10^6^) in the right flank area. (**D**) Changes in tumor volumes in xenograft mice injected with AF (n = 6) and AD tumorispheres (n = 5). Data are mean ± SEM and were analyzed using unpaired *t* test ***p < 0.001 between AF and AD.

Colony formation assay is one of the *in vitro* techniques that examine the ability of the cells to form colonies in semisolid agar. AF pituitary tumorispheres formed increased number of colonies in soft agar under normal growth factors as compared to AD pituitary tumorispheres (Fig. 6A). Determination of the percentage of the cells migrating through the pores in a Boyden chamber assay, which mimics the ability of the cells to penetrate the extra cellular matrix for invasion and metastasis, showed increased migration of AF pituitary tumorispheres than that of AD pituitary tumorispheres (Fig. 6B). The tumorigenic potential of the pituitary tumorispheres was studied by inoculating the AF and AD pituitary tumorisphere cells into immunodefecient mice. Pituitary tumorispheres of AF rats, but not of AD rats, successfully generated tumors in immunodeficient hosts, and these xenotransplanted tumors grew rapidly and formed large tumors in the hosts (Fig. 6C,D).

## Discussion

Pituitary tumors are the second most common intracranial tumor, accounting for approximately 20% of all types of the intracranial tumors. Around 40–60% of pituitary tumors are prolactinomas^16^. Although most pituitary tumors are benign and treatable, some tumors are invasive and aggressive and pose therapeutic challenges^27,28^. Even with the new surgical technique, radiological and MRI diagnoses, histological findings and some biochemical analyses, it is hard to detect the penetration of the tumor cells to the surrounding tissue such as dura, nasal cavity and diaphragm. Additionally, some pituitary tumors have genetic features which makes them prone to relapse and recur with more aggressive pattern or metastatic behavior^29,30^. The cause for the development of aggressive pituitary tumors is currently unknown. The data of the present study suggest that fetal alcohol exposure may be one of the environmental factor that promote the development of aggressive pituitary tumors.

Our conclusion that fetal alcohol-exposed rat develops aggressive pituitary tumors is based on anatomical, histological, biochemical evidence. Pituitary tumors of fetal alcohol-exposed offspring were large, hemorrhagic and appeared to penetration to the neighboring tissue. In human, some aggressive macroprolactinomas remain within the sella, while others extend beyond its confines and extensively invade the bones comprising the base of the skull^31^. Pituitary tumors of fetal alcohol-exposed offspring also show many immunohistochemical markers that characterize aggressive pituitary adenoma. For example, the Ki67 labeling index in pituitary tumors of fetal alcohol-exposed rats was >4%, which is in the range that diagnoses the pituitary invasiveness^32^. AF tumor also had P53 labeling index >30, which is within the labeling index range (>15%) that characterize invasive adenoma^33^. Also, the pituitary tumors of fetal alcohol exposed rats had elevated levels of oncogenes PTTG, FGF4 and MMP-9, which are known to be upregulated in the invasive prolactinoma and pituitary carcinoma^24,34,35^.

The data presented here connected elevated estrogen levels with the tumor promotion in the pituitary of fetal alcohol-exposed rats. We showed increased levels of pituitary aromatase and plasma estrogen in AF rats. Also, exogenous estrogen was more effective in increasing pituitary weight, plasma levels of PRL and tumor aggressiveness in the pituitary of AF rats. It has previously been shown that locally produced estrogen through aromatization might enhance tissue expression of *Pttg* and *Fgf* genes and promote aggressiveness of pituitary tumors^36^. It should be noted that heavy alcohol drinking in the adult promotes development of aggressive cancer in various tissues including mammary glands^37^. In the case of mammary gland, sex hormones, such as estrogen is also considered to play an important role in growth factors expression abnormalities and the etiology of alcohol promotion of aggressive cancer^38^. We therefore propose that elevated production of estrogen in the pituitary may be a cause for the development of aggressive pituitary tumors in fetal alcohol exposed rats.

The data presented here also implicates an increased stem cell niche within the tumor microenvironment with the tumor aggressiveness. AF tumors overexpressed multipotency proteins (SOX2, Oct4 and CD133). These proteins are established markers of pituitary stem cells^39^, are expressed in pituitary tumors^40^ and are known to be overexpressed in cells with high tumoriginicity^41^. A recent study also showed that forced up-regulation of the SOX2-positive pituitary stem cells by transgenic approaches in mouse stimulates a transient burst of proliferation, and subsequently induce tumorigenesis in a non-cell autonomous manner^42^. The pituitary tumors of fetal alcohol-exposed rats also developed tumor spheres in ultra-low attachment plate and expressed increased levels of stem cell marker proteins and genes, suggesting that pituitary tumors of AF rats have an increased stem cell niche. It is well known that cancer malignancy and metastatic ability of the tumor cells depends on the “stemness” phenotypes such as self-renewal, proliferation and migration features^43,44^. Recent evidence also suggest that cancer stem cells play an important role in alcohol-induced tumor initiation, progression and metastasis of breast and other cancers^45^. Our data showed that pituitary tumor cells of AF rats grew rapidly, showed high colony formation, cell migration, and successfully induced tumors in xenotransplanted mice. Together these data provide strong evidence to support that the pituitary gland of fetal alcohol exposed rat develops aggressive and invasive adenoma, possibly due to an increase in estrogenicity and an expanded stem cell niche within the tumor microenvironment. Further investigation is need to identified the molecular mechanism(s) underlying fetal alcohol promotion of aggressive prolactinomas.

## Methods

Animal surgery and care were performed in accordance with institutional guidelines and compiled with policies of the National Institutes of Health. Rutgers institutional animal care and use committee (IACUC) approved this research protocol. Fisher-344 rats were obtained from Harlan Laboratories (Indianapolis, IN) and housed under controlled condition of 12 h light/dark cycle at a constant temperature 22 °C throughout the study. Rats were housed in pairs in Open-type Shoe Box Cages with Bedcob bedding, and they were fed ad libitum with rat chow and tap water in a conventional facility. Health status of animals was checked regularly by determining body weight, feeding and general behaviors, and university veterinarians were consulted to address any special health needs. Some of these rats were bred, and on gestational day 7 through 21 they were fed either rat chow ad libitum (AD), a liquid diet containing ethanol (AF; Bioserve, Frenchtown NJ) ad libitum, or pair-fed (PF; Bioserve) an isocaloric liquid control diet (with ethanol calories replaced by maltose-dextrin). The concentration of ethanol in the diet was increased over the first 4 days from 1.7 to 5.0% v/v to habituate the animals to the alcohol diet. After this habituation period, rats were fed the liquid diet containing ethanol at a concentration of 6.7% v/v. We have previously shown that the liquid-diet paradigm in pregnant rats elevates the blood ethanol concentration in the range of 80–90 mg/dl at 2 h after the beginning of the dark period^22^. On postnatal day 2 (PD2), AF and PF pups were cross-fostered to untreated lactating AD dams to prevent any compromised nurturing by the AF and PF moms. Litter size was maintained at 8 pups per dam to minimize any nurturing effect on the body growth. Pups were weaned on PD21 and housed by sex. In each experimental group only one female offspring from a rat litter was used.

We determined whether fetal alcohol exposure changed pituitary lactotropic cell growth and hormone production during adulthood. For this, a group of AD, PF and AF female rats at 90 days of age on the day of estrus were sacrificed by decapitation (6 to 8 animals/feeding group). Pituitary tissues were collected for pituitary weight and protein and gene measurements, and plasma samples were obtained for PRL measurement. We also determined if fetal alcohol exposure altered the mitogenic effects of estrogen on lactotropes. For this, a group of animals at 60 days of age was ovariectomized under sodium pentobarbital anesthesia (50–60 mg/kg, i.p.) as a general anesthesia and 2.5% Bupivacaine (sc) as a local analgesia, and sc implanted with an estradiol-17β (Sigma, St. Louis, MO) filled 1-cm silastic capsule (Dow Corning, Midland, MI) or an empty 1-cm silastic capsule. These estradiol capsules were shown to maintain plasma levels of estradiol-17β between 120 and 150 pg/ml^46^. After surgery, animals were observed for pain and suffering or infection for 3 days. Estrogen-treated rats were kept in cages fitted with HEPA filter for prevention of estrogen contamination. At 120 days after the estradiol implants the rats were killed and the pituitary tissues were fixed for microscopic examination or frozen for further biochemical evaluation.

Fixed pituitary tissues were embedded in Paraplast and sectioned at 6 µm. Some of these sections were stained with hematoxylin and eosin then mounted with Permount. A toxicological pathologist, who was blind to treatment, viewed tumor slides to determine whether the tumors were adenomas or invasive adenoma. Representative images were taken using a Nikon photo microscope at 10X and 20X. Other sections were used for immunocytochemical detection of proteins. All the antibodies vendor and information is listed in Supplement Table 1. In general, tissue sections on a slide were blocked in normal horse serum (Vector, Burlingame, CA) for 60 minutes at room temperature. Sections were incubated overnight at 4 °C with either primary antibody or rabbit primary antibody isotype control (Life Technologies; Grand Island, NY), which served as a negative control. The following day the tissues sections were incubated with secondary antibody (Vector, Burlingame, CA) for 60 minutes at room temperature, counterstained with DAPI (Vector, SK-4100) then mounted with Permount prolong media. For all immunocytochemical detection, tumor sections were viewed and five representative images were captured at random from one section per tumor tissue using an Olympus FSX100 microscope at 20X (Olympus). The pictures were taken with the same exposure settings for all samples. The dark brown staining is counted as positive staining while the counter blue staining (nuclear staining) counted as negative staining. The percentage stained cells were calculated by dividing the number of positive stained cells with the number of negative stained cells multiply by 100.

Plasma PRL and estradiol levels were measured using rat PRL EIA kit (Alpco Diagnostics, Salem, NH) and rat estrogen ELISA kit (My BioSource, MBS703614) respectively, using the instructions from the manufacturer.

Expression levels of various genes in rat pituitaries were measured by quantitative real time PCR (SYBR green assay). Total RNA from the pituitary gland was extracted using the All in One Purification Kit (Norgen Biotek, Ontario, Canada). Total RNA (1 μg) was converted to first strand complementary DNA (cDNA) using a high capacity cDNA reverse transcription kit (Applied Biosystems, Carlsbad, CA). All the primer sequences used for the study are given in Supplemental Table 2. Real-time quantitative PCR was performed at 95 °C for 5 min followed by 40 cycles of 95 °C for 15 sec, 60 °C for 30 sec, 72 °C for 40 sec in an Applied Biosystems 7500 Real-time PCR system. The quantity of target gene expression was measured using standard curve method. *Gapdh* and ribosomal protein 19 (*Rpl*-19) were used as internal controls. Gene expression levels as GAPDH ratios are only presented in the figures. Gene expression levels as RPL-19 ratios are presented in Supplemental Fig. 1.

Data were analyzed using Prism 5.0 (GraphPad Software). The data shown in the figures are mean ± SEM. Significant differences between different treatment groups were assessed with one-way analysis of variance (ANOVA) with post-hoc analysis using the Newman Keuls posttest. P < 0.05 was considered significant. (See ‘Availability of materials and data’ section for more information)

### Data availability

Additional information on materials and data are available upon request.

## Electronic supplementary material

Supplementary Information

## References

[1] National Cancer Institute. Alcohol and cancer risk. https://www.cancer.gov/about-cancer/causes-prevention/risk/alcohol/alcohol-fact-sheet.

[2] Varela-Rey M, Woodhoo A, Martinez-Chantar ML, Mato JM, Lu SC (2013). Alcohol, DNA methylation, and cancer. Alcohol Res..

[3] Scoccianti C, Straif K, Romieu I (2013). Recent evidence on alcohol and cancer epidemiology. Future Oncol..

[4] Forsyth CB, Tang Y, Shaikh M, Zhang L, Keshavarzian A (2010). Alcohol stimulates activation of snail, epidermal growth factor receptor signaling, and biomarkers of epithelial-mesenchymal transition in colon and breast cancer cells. Alcohol. Clin. Exp. Res..

[5] Welsch T (2006). Update on pancreatic cancer and alcohol-associated risk. J. Gastroenterol. Hepatol..

[6] Papa NP (2017). Total and beverage-specific alcohol intake and the risk of aggressive prostate cancer: a case-control study. Prostate Cancer Prostatic. Dis..

[7] Hilakivi-Clarke L (2004). In utero alcohol exposure increases mammary tumorigenesis in rats. Br J Cancer..

[8] Polanco TA, Crismale-Gann C, Reuhl KR, Sarkar DK, Cohick WS (2010). Fetal alcohol exposure increases mammary tumor susceptibility and alters tumor phenotype in rats. Alcohol Clin. Exp. Res..

[9] Polanco TA, Crismale-Gann C, Cohick WS (2011). Alcohol exposure in utero leads to enhanced prepubertal mammary development and alterations in mammary IGF and estradiol systems. Horm. Cancer..

[10] Gomez F, Reyes FI, Faiman C (1977). Nonpuerperal galactorrhea and hyperprolactinemia. Clinical findings, endocrine features and therapeutic responses in 56 cases. Am. J. Med..

[11] Garcia MM, Kapcala LP (1995). Growth of a microprolactinoma to a macroprolactinoma during estrogen therapy. J Endocrinol. Invest..

[12] Wiklund J, Wertz N, Gorski J (1981). Acomparison of estrogen effects on uterine and pituitary growth and prolactin synthesis in F344 and Holtzman rats. Endocrinology..

[13] Sarkar DK, Gottschall PE, Meites J (1982). Damage to hypothalamic dopaminergic neurons is associated with development of prolactin-secreting pituitary tumors. Science..

[14] Lloyd RV (1983). Estrogen-induced hyperplasia and neoplasia in the rat anterior pituitary gland. An immunohistochemical study. Am. J. Pathol..

[15] Colao A (2009). Pituitary tumours: the prolactinoma. Best Pract. Res. Clin. Endocrinol. Metab..

[16] Dolecek TA, Propp JM, Stroup NE, Kruchko C (2012). CBTRUS statistical report: primary brain and central nervous system tumors diagnosed in the United States in 2005–2009. Neuro-Oncology..

[17] Roelfsema F, Biermasz NR, Pereira AM (2012). Clinical factors involved in the recurrence of pituitary adenomas after surgical remission: a structured review and meta-analysis. Pituitary..

[18] Banik A (2017). Maternal factors that induce epigenetic changes contribute to neurological disorders in offspring. Genes (Basel).

[19] Veazey KJ, Parnell SE, Miranda RC, Golding MC (2015). Dose-dependent alcohol-induced alterations in chromatin structure persist beyond the window of exposure and correlate with fetal alcohol syndrome birth defects. Epigenetics Chromatin..

[20] Sarkar DK (2016). Male germline transmits fetal alcohol epigenetic marks for multiple generations: a review. Addict. Biol..

[21] Gangisetty O, Wynne O, Jabbar S, Nasello C, Sarkar DK (2015). Fetal alcohol exposure reduces dopamine receptor D2 and increases pituitary weight and prolactin production via epigenetic mechanisms. PLoS One..

[22] Gangisetty O, Jabbar S, Wynne O, Sarkar DK (2017). MicroRNA-9 regulates fetal alcohol-induced changes in D2 receptor to promote prolactin production. J. Endocrinol..

[23] Langlois F, McCartney S, Fleseriu M (2017). Recent progress in the medical therapy of pituitary tumors. Endocrinol. Metab. (Seoul)..

[24] Mete O, Ezzat S, Asa SL (2012). Biomarkers of aggressive pituitary adenomas. J. Mol. Endocrinol..

[25] Manoranjan B (2016). The identification of human pituitary adenoma-initiating cells. Acta Neuropathol. Commun..

[26] Carreno G, Gonzalez-Meljem JM, Haston S, Martinez-Barbera JP (2017). Stem cells and their role in pituitary tumorigenesis. Mol. Cell. Endocrinol..

[27] Selman WR, Laws ER, Scheithauer BW, Carpenter SM (1986). The occurrence of dural invasion in pituitary adenomas. J. Neurosurg..

[28] Meij BP, Lopes MB, Ellegala DB, Alden TD, Laws ER (2002). The long-term significance of microscopic dural invasion in 354 patients with pituitary adenomas treated with transsphenoidal surgery. J. Neurosurg.

[29] Partington MD, Davis DH, Laws ER, Scheithauer BW (1994). Pituitary adenomas in childhood and adolescence. Results of transsphenoidal surgery. J. Neurosurg..

[30] Lillehei, K. O., Kirschman, D. L., Kleinschmidt-DeMasters, B. K. & Ridgway, E. C. Reassessment of the role of radiation therapy in the treatment of endocrine-inactive pituitary macroadenomas. *Neurosurger*y, **4**3, 432–438; discussion 438–439 (1998). 10.1097/00006123-199809000-00020 9733298

[31] Scheithauer BW, Kovacs KT, Laws ER, Randall RV (1986). Pathology of invasive pituitary tumors with special reference to functional classification. J. Neurosurg..

[32] Zhao D, Tomono Y, Nose T (1999). Expression of P27kip1 and Ki-67 in pituitary adenomas: an investigation of marker of adenoma invasiveness. Acta. Neurochir (Wien).

[33] Thapar, K., Scheithauer, B. W., Kovacs, K., Pernicone, P. J. & Laws, E. R. Jr. p53 expression in pituitary adenomas and carcinomas: correlation with invasiveness and tumor growth fractions. *Neurosurgery*. **38**, 765–770; discussion 770–771 (1996). 8692397

[34] McCabe CJ (2003). Expression of pituitary tumour transforming gene (PTTG) and fibroblast growth factor-2 (FGF-2) in human pituitary adenomas: relationships to clinical tumour behaviour. Clin Endocrinol (Oxf)..

[35] Kawamoto H (1996). Matrix metalloproteinase-9 secretion by human pituitary adenomas detected by cell immunoblot analysis. Acta Neurochir (Wien)..

[36] Ozkaya HM (2016). Locally produced estrogen through aromatization might enhance tissue expression of pituitary tumor transforming gene and fibroblast growth factor 2 in growth hormone-secreting adenomas. Endocrine..

[37] Wang Y, Xu M, Ke ZJ, Luo J (2017). Cellular and molecular mechanisms underlying alcohol-induced aggressiveness of breast cancer. Pharmacol Res..

[38] Dumitrescu RG, Shields PG (2005). The etiology of alcohol-induced breast cancer. Alcohol..

[39] Chang CV (2017). Differential expression of stem cell markers in human adamantinomatous craniopharyngioma and pituitary adenoma. Neuroendocrinology..

[40] Gao Z (2017). Expression of stem cell markers and dopamine D2 receptors in human and rat prolactinomas. Med. Sci. Monit..

[41] Yi XJ (2015). Aberrant Wnt/β-catenin signaling and elevated expression of stem cell proteins are associated with osteosarcoma side population cells of high tumorigenicity. Mol. Med. Rep..

[42] Andoniadou CL (2013). Sox2(+) stem/progenitor cells in the adult mouse pituitary support organ homeostasis and have tumor-inducing potential. Cell Stem Cell..

[43] Krivtsov AV (2006). Transformation from committed progenitor to leukaemia stem cell initiated by MLL-AF9. Nature..

[44] Somervaille TC, Cleary ML (2006). Identification and characterization of leukemia stem cells in murine MLL-AF9 acute myeloid leukemia. Cancer Cell..

[45] Xu M, Luo J (2017). Alcohol and Cancer Stem Cells. Cancers (Basel)..

[46] De. A, Boyadjieva N, Pastorcic M, Sarkar DK (1995). Potentiation of estrogen’s mitogenic effect on the pituitary gland by alcohol consumption. Int. J. Oncol..

