# Supplementary material for: Prenatal alcohol exposure increases the susceptibility to develop aggressive prolactinomas in the pituitary gland
Source: Sci Rep. 2018 May 16;8:7720. doi: 10.1038/s41598-018-25785-y (PMC5955957; doi:10.1038/s41598-018-25785-y)

Sarkar, Supplemental Table 1.

Table 1. Information about antibodies and their vendors

Antibody	Cat number	Vendor information
Ki67	Abcam-15580	Abcam, Cambridge, MA
P53	Abcam, PAb 240	Abcam, Cambridge, MA
PTTG	PTTG Antibody (H-160)	Santa Cruz, Santa Cruz, CA
FGF4	SC, 9006	Santa Cruz, Santa Cruz, CA
MMP9	Ab38898	Abcam, Cambridge, MA
SOX2	MBS462135	MyBiosource, San Diego, CA
Nanog	ab106465	Abcam, Cambridge, MA
CD44	Ab 5640s	Abcam, Cambridge, MA
KLF4	Anti-KLF4 (ab72543)	Abcam, Cambridge, MA
CD133	anti-CD133 antibody	MyBiosource, San Diego, CA
TSH	AB976	Millipore, Dresher, PA
Growth hormone	BAF1566	R&D system, Minneapolis, MN
Prolactin	AF1112	R&D system, Minneapolis, MN

Sarkar Supplemental Table 2.

Table 2. Primer sequences

Primers	Sequences
FGF4 Forward	ACATCGTTATCAACGGCAGC
FGF4 Reverse	GTCTTCTTCCTCTGCTTCGG
PTTG Forward	GTGCCAACATCAACAAACGA
PTTG Reverse	GCATTGAGGAAGGCTGGAAGA
MMP9 Forward	CGGATCCCCCAACCTTTACC
MMP9 Reverse	AGGTCAGAACCGACCCTACA
αESR1 Forward	TCGGGAATGGCCTTGTTG
αESR1 Reverse	AGCTGCGGGCGATTGA
SOX2 Forward	AGAACTAGACTCCGGGCGAT
SOX2 Reverse	ACCCAGCAAGAACCCTTTCC
Nanog Forward	TGCATTTGTCTGAGCTGGGTA
Nanog Reverse	TGGTATGGAGTAGGGTGGGT
OCT4 Forward	GGGGACATCTTGGGTTGGAG
OCT4 Reverse	AGTAGAGCAGTGGGGGTAGG
KLF4 Forward	TGTGACTATGCAGGCTGTGG
KLF4 Reverse	GTGTGGGTCATGTCCACGAT
Sca1	TGAGGATGGACACTTCTCACAC
Sca1	GAACATTGCAGGACCCCAGA
CD34 Forward	AGGTTAGGCCCGAGTGTTTG
CD34 Reverse	TAAGGGTCTTCACCCAGCCT
Nestin Forward	CTGTGGGTGTCAGTGGTCTC
Nestin Reverse	TTAGAGCACCCACCTCCTGT
S100 Forward	AGCTTCTCTGTCTACCCTCCT
S100 Reverse	TCTTCGTCCAGCGTCTCCAT
Pit-1 Forward	CTGTGGTAGCCATGTGTGGT
Pit-1 Reverse	TATTCACATATATGATGGCCTCTCT
CD44 Forward	CTACCCCTGAAACACCACCC

CD44 Reverse	TTAGCGCCGCTCTTAGTGCT
CD133 Forward	ACCAAGGAGGTCGCCATCTA
CD133 Reverse	CGAGTCCTTGTCTGCTGGTT
PRL Forward	ACCGTGTGGTCATGCTTTCT
PRL Reverse	AGCCGCTTGTTTTGTTCCTC

Sarkar, Supplement Fig. 1

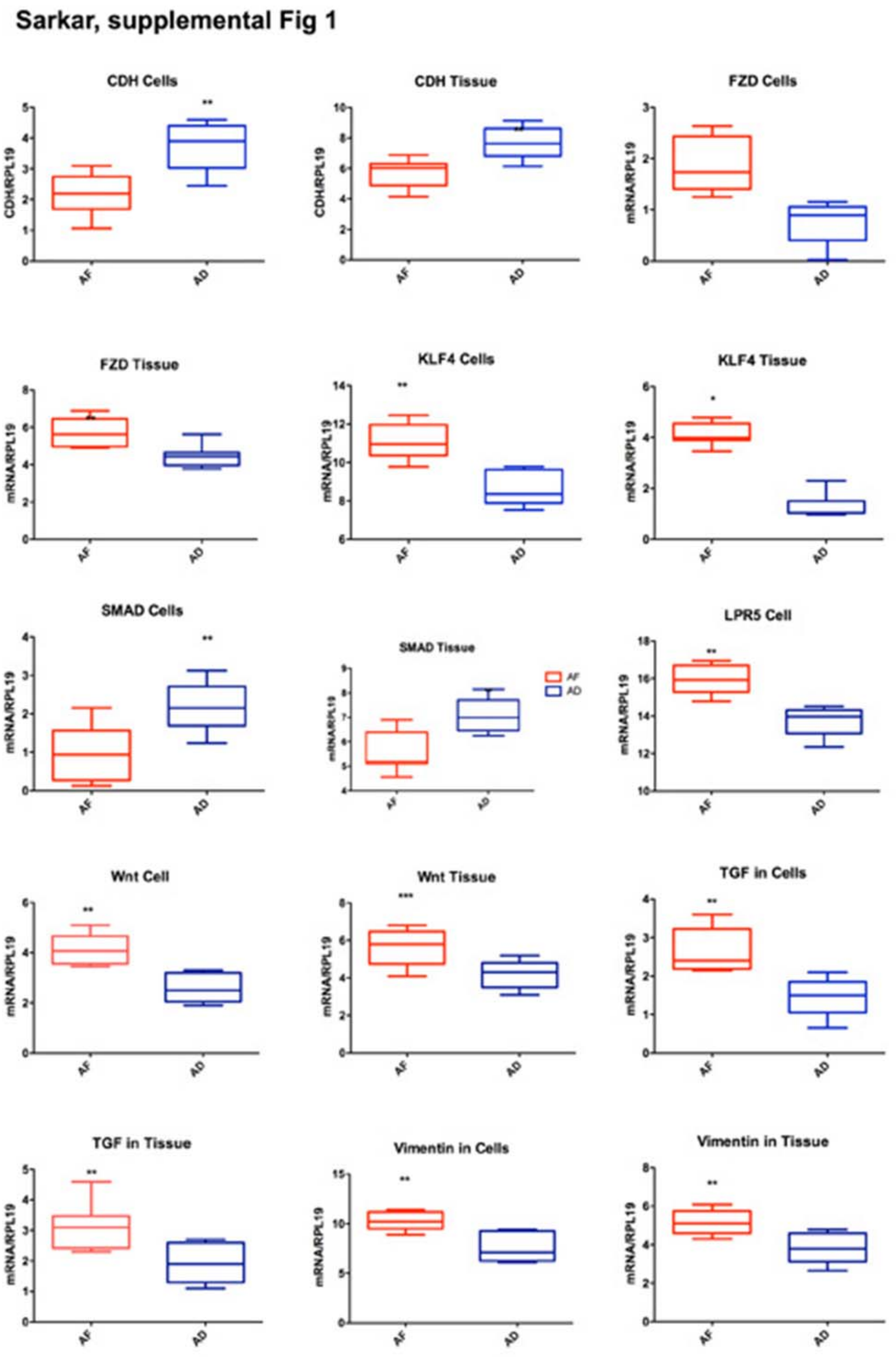

Supplement: Supplementary file 1 — Supplementary Information [file 41598_2018_25785_MOESM1_ESM.pdf]
